# Cancer-Chemotherapy-Related Regimen Checks Performed by Pharmacists of General Hospitals Other than Cancer Treatment Collaborative Base Hospitals: A Multicenter, Prospective Survey

**DOI:** 10.3390/pharmacy12010001

**Published:** 2023-12-20

**Authors:** Daisuke Ueki, Shinya Suzuki, Takahiro Ohta, Akira Shinohara, Yasukata Ohashi, Daisuke Konuma, Yasuaki Ryushima, Ryoko Udagawa, Hironori Motoshige, Masahiro Ieoka, Akihiro Taji, Yuuki Kogure, Mikako Hiraike, Miyuki Uoi, Kazuhiko Ino, Toshikatsu Kawasaki, Masakazu Yamaguchi

**Affiliations:** 1 Department of Pharmacy, National Hospital Organization Tokyo National Hospital, Tokyo 204-8585, Japan; 2 Department of Pharmacy, National Cancer Center Hospital East, Chiba 277-8577, Japan; 3 Department of Pharmacy, National Hospital Organization Tokyo Medical Center, Tokyo 152-8902, Japan; 4 Department of Pharmacy, National Center for Global Health and Medicine, Tokyo 162-8655, Japan; 5 Department of Pharmacy, National Hospital Organization Chiba Medical Center, Chiba 260-8606, Japan; 6 Department of Pharmacy, National Hospital Organization Saitama Hospital, Saitama 351-0102, Japan; 7 Department of Pharmacy, National Cancer Center Hospital, Tokyo 104-0045, Japan; 8 Department of Pharmacy, National Hospital Organization Hokkaido Medical Center, 5-7 Yamanote, Nishi-ku, Sapporo 063-0005, Japan; 9 Department of Pharmacy, National Hospital Organization Hamada Medical Center, Matsue 697-8511, Japan; 10 Department of Pharmacy, National Hospital Organization Osaka Minami Medical Center, Osaka 586-8521, Japan; 11 Department of Pharmacy, National Hospital Organization Higashihiroshima Medical Center, Hiroshima 739-0041, Japan; 12 Department of Pharmacy, National Hospital Organization Kumamoto Medical Center, Kumamoto 860-0008, Japan; 13 Department of Pharmacy, National Hospital Organization Kyusyu Cancer Center, Fukuoka 811-1395, Japan; 14 Department of Pharmacy, Cancer Institute Hospital, Tokyo 135-8550, Japan

**Keywords:** cancer chemotherapy, chemotherapy regimen checks, pharmacist intervention, hospital pharmacist

## Abstract

Although prescription review is an important role for pharmacists in anticancer drug therapy, there are no guidelines in Japan that specify what pharmacists should check for in chemotherapy regimens. This prospective multicenter survey aimed to investigate the implementation of chemotherapy regimen checks by pharmacists in general hospitals by focusing on 19 recommended confirmation items designed to enhance chemotherapy safety. This study involved 14 hospitals within the National Hospital Organization in different regions of Japan. The top five cancers in Japan (gastric, colorectal, lung, breast, and gynecological) were targeted and specific chemotherapy regimens were analyzed. This study assessed the amount of time required for regimen checks, the number of confirmation items completed, the number and the content of inquiries raised regarding prescriptions, and the pharmacists’ opinions using a questionnaire that had a maximum score of 10 points. Pharmacists checked 345 and 375 chemotherapies of patients in the control group (CG) and recommended items group (RIG), respectively. The mean time periods required for completing a chemotherapy regimen check were 4 min and 14 s (SD ±1 min and 50 s) and 6 min and 18 s (SD, ±1 min and 7 s) in the CG and RIG, respectively. The mean of the recommended items for the CG = 12.4 and for the RIG = 18.6. The items that the pharmacists did not confirm included urine protein (sixty-nine cases, 18.4%), allergy history (four cases, 1%), previous history (two cases, 0.5%), and a previous history of hepatitis B virus (sixty-nine cases, 18.4%). The number of inquiries for a doctor’s prescription order was higher in the RIG than in the CG (41 vs. 27 cases). This multicenter survey demonstrated the potential effectiveness of implementing 19 recommended confirmation items in the regimen checks by pharmacists in general hospitals other than cancer treatment collaborative base hospitals.

## 1. Introduction

Remarkable progress and advancements in the field of cancer chemotherapy have led to significant improvements in the survival outcomes of patients with cancer. Although a wide variety of treatment options are now available through the release of molecular targeting drugs and new modalities, the safe management of cancer chemotherapy persists as an issue requiring intervention [1,2]. According to a Norwegian study, out of 3557 participants, a total of 3372 medication errors occurred during administration and the leading types of errors were dosing (38%), omissions (23%), and wrong drugs (15%) [3]. The therapeutic dosage of cytotoxic anticancer drugs is generally narrow because increasing the dosage increases their toxicity. Their dosing intervals are also strictly defined in the package inserts. Therefore, such errors in cancer chemotherapy cause life-threatening adverse drug events and several measures have been implemented in each hospital [4,5]. To minimize dosing errors, guidelines for cancer chemotherapy regimens were released in 1998 [6]. However, the guidelines are limited to proposing standard rules for describing chemotherapy regimens. In Japan, hospitals use a standardized chemotherapy regimen order set; instead of ordering prescriptions one by one, a comprehensive set of anticancer drugs, antiemetics, and supplemental solutions are assembled to eliminate cancer-chemotherapy disparities. In addition to doses of anticancer drugs, regimens include several aspects, such as supportive medications and rest periods. Regimens largely contribute to ensuring medical safety and standardizing cancer chemotherapy. A chemotherapy regimen order is mandated for the safe prescription of anticancer drugs and their administration [7]. Ranchon et al. analyzed a total of 91 errors and proposed 34 corrective actions in 10 combined reviews using a systematic approach combining data from antineoplastic medication error reviews and morbidity and mortality conferences to reduce the occurrence of medication errors; however, the details were not simple. The response required the involvement of multiple disciplines, as well as physicians [8]. These analyses indicate that the confirmation of medications prescribed by healthcare professionals other than physicians is essential. Pharmacists are responsible for checking physicians’ prescriptions and improving inappropriate prescribing in clinical practice [9,10,11]. Although prescription review plays an important role for pharmacists in anticancer drug therapy, there are no guidelines in Japan that specify what pharmacists should check for in chemotherapy regimens.

Intervention by pharmacists in cancer chemotherapy orders written by physicians prevents dosing errors [12,13,14]. In Japan, such duties of pharmacists are referred to as chemotherapy regimen checks or a pharmacist’s evaluation of a doctor’s chemotherapy regimen order [15,16]. However, to the best of our knowledge, no study has defined the selection criteria for the confirmation items of regimen checks. Therefore, a difference in the quality of medical resources and pharmacists’ competence may affect the successful implementation of regimen checks. Regarding confirmation items of regimen checks, Suzuki et al. performed a study involving pharmacists at cancer-designated hospitals and voluntarily surveyed some of the items [17]. Griffin et al. reported 57 systematic checks used for parenteral anticancer drugs [18]; however, they did not define the selection criteria for confirmation items. Using this background, Ohta et al. prepared and used a questionnaire to examine confirmation items recommended for regimen checks [19]. The results yielded 19 recommended items, which comprised items that are commonly confirmed via pharmacists and those that the authors determined to be essential for regimen checks, regardless of the frequency at which checks are performed by pharmacists (Table 1). 

The confirmation of all 19 items is ideal; however, performing regimen checks increases the workload. Furthermore, for some institutions, it might be difficult to perform the confirmation of some items owing to the absence of medical systems/resources. The aim of this prospective study was to examine the feasibility and difficulties of confirming 19 chemotherapy regimen checklist items in daily hospital pharmacy practice to evaluate the possibility of their application to clinical practice.

## 2. Materials and Methods

### 2.1. Institutions Analyzed

The National Hospital Organization is a Japanese hospital organization comprising 6 groups according to geographical areas and managing 140 institutions. This prospective survey was performed on pharmacists from 14 hospitals (2 hospitals from the “Hokkaido/Tohoku group”, 5 hospitals from the “Kanto/Shinetsu group”, 2 hospitals from the “Tokai/Hokuriku group”, 2 hospital from the “Kinki group”, 1 hospital in the “Chugoku/Shikoku group”, and 2 in the “Kyusyu group”) to eliminate intra- and intergroup bias (Table 2). Among the general hospitals willing to participate in this study, we included those whose number of claims for additional outpatient cancer chemotherapy was ≥500/year. Base hospitals were excluded if they had fewer than two of the four categories of hospital departments, considering the chemotherapy regimen being studied: respiratory medicine, gastroenterology, surgery, and gynecology. 

### 2.2. Selected Chemotherapy Regimens Investigated in This Study

The top 5 most frequent cancers in Japan (i.e., gastric, colorectal, lung, breast, and gynecological) were targeted. The most commonly used regimens reported in the preliminary questionnaire were analyzed. These included S-1 + oxaliplatin (SOX) (gastric cancer), FOLFIRI + bevacizumab (Bev) (colorectal cancer), pembrolizumab/carboplatin + pemetrexed (CBDCA + PEM) (lung cancer), epirubicin + cyclophosphamide (EC) (breast cancer), and carboplatin + paclitaxel (TC) (gynecological cancer). In lung cancer, the number of pharmacists who dispense these two drug regimens was the same as reported in the preliminary questionnaire; therefore, these two chemotherapy regimens, pembrolizumab and CBDCA + PEM, were investigated in this study. 

### 2.3. Investigation Methods

#### 2.3.1. Measuring the Time Required for the Regimen Check Service 

We investigated the duties of the pharmacists when they performed regimen checks on inpatients and outpatients according to the cancer type between 1 July 2019, and 30 September 2019. This prospective study was a pre- and postimplementation comparison of 19 chemotherapy regimen checklist items by the same pharmacist. Between 1 July and 12 August, pharmacists were asked to perform regimen checks using hospital-specific confirmation items and this constituted the control group. Between 13 August and 30 September, the pharmacists in the control group were instructed to perform regimen checks using the recommended items (Table 1) and this constituted the recommended items group. The same stopwatch was used to measure the time required to complete the regimen checks by the two groups. The working time was measured by the pharmacists themselves, who performed the work and used a stopwatch that was purchased for this study, and distributed to each hospital. Since the control group and the recommended items group were surveyed before and after the introduction of the chemotherapy regimen, the dates of the check and those of the patients checked are different. The regimen checking process performed by the pharmacists was the same in both groups; however, 19 items were checked in the recommended items group, which added to the work.

#### 2.3.2. Checklist Items That Could and Could Not Be Confirmed among the Recommended Items

The numbers of items that were confirmed in the regimen check were tabulated, as well as the items that could not be confirmed.

#### 2.3.3. Number and Contents of Inquiry for Doctors’ Prescription Orders

We tabulated the contents of the inquiry for a doctor’s prescription order that arose from the regimen checks performed by the pharmacists. 

#### 2.3.4. Questionnaire 

The questionnaire was administered to the group at the end of the first phase, constituting the control group, and at the end of the second phase, as the recommended items group. The questionnaire, which consisted of a maximum score of 10 points (0–10 points), was administered to the pharmacists who performed regimen checks. The pharmacists in the recommended items group were asked whether the recommended items were potentially applied in clinical practice. The scoring was such that a score of 0 was more negative and a score of 10 meant more positive. The scale for the potential clinical application of the recommended items was rated on a scale of 0 to 10 points per question, relative to that pharmacist’s impression of the item as positive or negative. The contents of the questionnaire are presented in Table 3.

### 2.4. Statistical Analysis

All statistical analyses were performed using EZR ver1.55 (Saitama Medical Center, Jichi Medical University, Saitama, Japan), which is a graphical user interface for R (The R Foundation for Statistical Computing, Vienna, Austria). More precisely, it is a modified version of R commander designed to add statistical functions and it is frequently used in biostatistics [20]. A paired *t*-test was used for comparing questionnaire scores; a *p*-value of less than 0.05 was considered to indicate statistical significance. Descriptive statistics were used to analyze the means, medians, and frequencies.

## 3. Results

### 3.1. Time Required for Completing Regimen Checks

The numbers of regimen checks performed were 345 and 375 in the control group and recommended items group, respectively. The mean time periods required for completing a regimen check (±standard deviation) were 4 min and 14 s (±1 min and 50 s) and 6 min and 18 s (±1 min and 7 s) in the control group and recommended items group, respectively. In the B and C Hospitals (Kanto/Shinetsu group), where electronic medical records had not yet been introduced and data on the prescription of anticancer drugs were recorded on paper, the mean time periods required for the completion of a regimen check were 4 min and 24 s (B Hospital) and 3 min and 10 s (C Hospital) in the control group, as compared to 9 min and 3 s (B Hospital) and 7 min and 24 s (C Hospital) in the recommended items group. The mean amount of time required for completing a regimen check was tabulated according to the regimen, a previous history of cancer chemotherapy, cycles of chemotherapy, and certification of cancer-related associations. The results show that a longer amount of time was needed for all items in the recommended items group (Table 4). 

### 3.2. Number of Confirmation Items Performed in Regimen Checks 

Among the 19 recommended items on the chemotherapy regimen checklist, the mean numbers of items that pharmacists confirmed were 12.4 and 18.6 in the control and recommended items groups, respectively. The items that pharmacists did not confirm were urine protein (sixty-nine cases, 18.4%), an allergy history (four cases, 1%), a previous history (two cases, 0.5%), and a previous history of hepatitis B virus (sixty-nine cases, 18.4%). 

### 3.3. Number and Contents of Pharmacist’s Inquiry for Prescriptions 

The number of inquiries for a doctor’s prescription order was higher in the recommended items group than in the control group (41 vs. 27 cases). The number of inquiries for a doctor’s prescription order related to dosage, premedication, urinalysis, and a history of hepatitis B virus was higher in the recommended items group than in the control group (Table 5).

### 3.4. Changing Attitudes toward Chemotherapy Regimen Checks

The respondents were the same 62 pharmacists classified into the control and recommended items groups (62 pharmacists for each group). The response rate was 100%. Regarding the question, “Are current regimen checks conducted by pharmacists useful for performing safe cancer chemotherapy?”, the scores of the recommended items group showed a significant increase (*p* < 0.05) (7.2 points in the control group vs. 8.2 points in the recommended items group). Regarding the question, “Do you have confidence in current regimen checks performed by pharmacists?”, the scores of the recommended items group significantly increased (*p* < 0.05) (5.4 points in the control group vs. 6.2 points in the recommended items group). Pharmacists were also asked to answer questions relating to the recommended items regimen check, such as “Did a recommended item affect the time required for completing a regimen check?”. They responded with “More time was required because the number of confirmation items increased” (44 pharmacists); “The number of confirmation items increased but the time did not change” (15 pharmacists); “The number of confirmation items increased but the amount of time was shortened” (0 pharmacists); and “The recommended items were not used because I did not understand them” (3 pharmacists). Regarding the question, “Is the distribution of the recommended items in hospitals in Japan meaningful?”, they responded that “It is meaningful for the equalization of knowledge and methodology in cancer care” (34 pharmacists); “It is meaningful because there is no definition for regimen checks at my institution; although, I may not utilize them” (8 pharmacists); “Further careful examination of the confirmation items is required for obtaining the maximal benefit from them” (20 pharmacists); and “It is not meaningful because I am unable to use them” (0 pharmacists).

## 4. Discussions

In the present study, we created 19 items that should be confirmed in regimen checks and examined the possibility of their application in clinical practice. Pharmacists in the recommended items group confirmed almost all items, with a mean of 18.6 items. The mean time required for completing a regimen check was only 2 min and 4 s longer in the recommended items group than in the control group, suggesting that the potential application of the 19 recommended items in clinical practice in general hospitals other than cancer-designated hospitals was high. Items that were not confirmed via numerous pharmacists were urinalysis and a history of hepatitis B virus. Given that the risk of proteinuria may increase after the use of molecular targeting drugs, such as bevacizumab, the confirmation of urine protein levels is essential. Furthermore, confirmation of urinalysis is highly recommended because type-1 diabetes secondary to immunotherapy is reported. The confirmation of a history of hepatitis B virus is important because anticancer drugs, primarily rituximab, cause reactivation of this disease, which has serious consequences [21]. The following theorizes the reason for such unsuccessful confirmation. Examination orders are required to confirm urinalysis and a history of hepatitis B virus; however, in general, in hospitals that participated in this study, many treatments were provided by physicians without special certification in cancer chemotherapy [22]. Thus, they were less likely to recognize the importance of examining the two items. The 19 recommended items were created based on items used in cancer-designated hospitals. This solid evidence may assist pharmacists in explaining their decision to physicians after they identify an error in the physician’s prescription orders in regimen checks using the 19 recommended items.

In terms of work burden, the mean time required for completing a regimen check was 2 min and 4 s longer in the recommended items group than in the control group. More than 70% of the pharmacists responded that more time was required to complete a regimen check because of the increased number of confirmation items. Such a response raises concerns regarding the potential application of the 19 recommended items to institutions with insufficient pharmacists. As a measure against such a burden on pharmacists, detailed data on items confirmed via pharmacists should be recorded after they perform regimen checks on patients who receive initial cancer chemotherapy. The difference in time required for completing a regimen check was compared between patients who received initial treatment and those who underwent second and subsequent treatments. The results showed that it was 1 min and 22 s in the recommended items group and 2 min and 22 s in the control group. This one-minute difference between the two groups suggested that pharmacists needed to confirm many items, even in patients in the control group, if they were cancer-chemotherapy-naïve patients.

Among the recommended items, data related to certain items, including an allergy history, were duplicated if pharmacists recorded them after initial regimen checks. This may reduce the time required for completing a regimen check in the second and subsequent treatments. In addition to regimen checks, hospital pharmacists in Japan are responsible for several duties, such as preparing anticancer drugs and dosing instructions. With this background, performing regimen checks is challenging for the same pharmacist each time. The time required for completing a regimen check is expected to be shorter if pharmacists who perform regimen checks document detailed data on cancer-chemotherapy-naïve patients in the patient’s chart or papers. 

Intergroup comparisons were performed to measure the difference in time required for completing a regimen check in the two institutions in which electronic medical records were not introduced. The results showed that the recommended item group spent 4 min 39 s (4 min 24 s vs. 9 min 3 s) and 4 min 14 s (3 min 10 s vs. 7 min 24 s) longer than the control group in hospitals B and C, which did not implement electronic medical records and prescribed anticancer drugs with paper prescriptions, respectively. These results were two minutes longer than the mean time of all institutions. This difference in time may be attributable to a problem related to systems. Information on the recommended items should be obtained from the patient’s chart, thereby inevitably requiring more time in institutions in which electronic medical records have not been introduced. The percentage of hospitals that have introduced electronic medical records has been increasing every year. However, according to a survey of medical institutions in Japan conducted by the Ministry of Health, Labour, and Welfare in 2020, this percentage was 57.2%, suggesting that many hospitals continue to use paper-based records [23]. In institutions without electronic medical records, the efficacy of regimen checks may be improved if pharmacists who perform regimen checks cooperate with hospital pharmacists who check the patients’ charts every day and ward nurses; although, only the physician’s contact information was given in this study. Technology plays an essential role in today’s healthcare environment [24,25]. The lack of electronic charting in some institutions, likewise, has a negative impact on generalizability [26].

The number of inquiries for a doctor’s prescription order was higher in the recommended items group than in the control group (41 cases vs. 27 cases). An increase in the number of confirmation items may be associated with safe cancer chemotherapy. The results of our questionnaire support this hypothesis. Specifically, the scores for the questions related to the usefulness of and confidence in the current regimen checks were higher in the recommended items group than in the control group. Furthermore, the percentage of pharmacists who positively responded to the question (i.e., those who answered that recommended items are meaningful for equalizing and defining the knowledge and methodology on cancer chemotherapy) was 68%, indicating that the recommended items will be distributed to many institutions in Japan. 

This study consisted of two stages, with each stage lasting for approximately 1.5 months. Thus, the learning curve of the pharmacists who perform regimen checks was not taken into consideration, which is a limitation of this study. If regimen checks using the recommended items are continued for 6 months or 1 year, the accuracy of the inquiry for a doctor’s prescription order is expected to increase. The results of our study with a duration of 1.5 months may be insufficient. However, this study aimed to evaluate whether the recommended items were applied in clinical practice. The results of this study suggest the possibility of their application, which is significant. 

We instructed the pharmacists to confirm only the recommended items when they performed regimen checks. We did not give sufficient attention to their knowledge of each anticancer drug and regimen. This indicates that pharmacists with insufficient expertise may not effectively use the recommended items. According to a report by Weiss et al., [4] the association of pharmacists’ knowledge with the successful implementation of regimen checks becomes strong in a complex regimen (i.e., several drugs are combined). The development of methods according to the complexity of the regimens is required. Our questionnaire shows that 32% of the pharmacists responded that the contents of the regimen checks should be scrutinized before the concept of regimen checks is standardized in Japan. A chemotherapy regimen check means checking physicians’ orders for anticancer drug therapy; this survey was designed to investigate the items to be checked. The chemotherapy regimens covered in this survey were relatively simple regimens to check rather than the long and complex regimens for hematologic malignancies. It is clear that the checking of such regimens requires prior knowledge of a wide variety of anticancer drugs. A study by Ohta et al. shows that the most common source of information used for regimen checks is package inserts [19]. However, finding information on regimen checklist items used as criteria for administration and required for expertise, such as supportive medications and laboratory values, is challenging only in package inserts. Package inserts are not designed to check the use of the drug. In addition, since anticancer drug therapy is performed in combination with several anticancer drugs, package inserts are very poor at describing cases in which the drugs are used in combination. Therefore, it is naturally difficult to check the regimen on the package insert. Information sources other than package inserts in Japan include interview forms released by pharmaceutical companies, a guide for the appropriate use of medication, the risk management plan, and specialized books released from cancer treatment collaborative base hospitals [27]. When the recommended items are introduced into each institution, using these useful materials and package inserts is recommended to establish a detailed tool for the checks according to the regimen.

Although six groups from the National Hospital Organization were incorporated into the present study, the results were composed of only fourteen facilities. Therefore, it is our understanding that we could not completely prevent bias because the number of anticancer agents and the number of pharmacists varied in each facility; this was true even in general hospitals other than cancer treatment base hospitals and other hospitals. In addition, although the survey targeted regimens with high usage rates among the five cancers with the highest incidence rates in Japan, taking the recent increase in the combination of immune check inhibitors and cytotoxic anticancer drugs into consideration is recommended. Furthermore, we did not account for the patients’ background; thus, it is possible that regimen checks were performed at some facilities on patients with a high history or concomitant medications. Considering the limitations of these research backgrounds, increasing the number of facilities and studying the latest treatment details is necessary. To accumulate the evidence of the 19 recommended items and create Japanese guidelines for regimen checks, we will distribute these items as a manual to hospitals of the National Hospital Organization that perform cancer chemotherapy. Furthermore, since this is a before-and-after comparative study using the same pharmacists’ data, there is the possibility of shift bias. Chemotherapy regimen checks only focus on the safety management of doctors’ prescriptions; however, adverse drug management is also an important role of pharmacists [28]. Optimal treatment practice naturally requires not only regimen checks but also comprehensive interventions, ranging from outpatient consultations to home telephone follow-ups [29]. Also, although this survey focused on regimen checks for intravenous anticancer drugs, the frequency of regimen checks for oral anticancer drugs, which are currently increasing in many areas, is lower than that for injectable anticancer drugs administered in hospitals; therfore, ensuring the safety of these drugs is also a future problem [30].

In this study, we developed 19 items to be included in regimen checks and examined their applicability in clinical practice. As of the end of 2023, all National Hospital Organization hospitals in Japan still performed chemotherapy regimen checks; however, the required checklist items have not been clarified. Our data will be used to help clarify the tasks and to develop a manual for the National Hospital Organization in the future.

## Figures and Tables

**Table 1:** Nineteen recommended confirmation items.

	Items	Additional Notes
1	Height	Confirm measurement date (if there is an institution-specific rule, e.g., within the last several months, confirm whether the measurement is performed within that period)
2	Weight	
3	Body surface	
4	Indications	Confirm cancer types for preventing an off-label regimen use
5	Regimens	Confirm the main purpose of a regimen (neoadjuvant therapy, adjuvant therapy, life-prolonging treatment, or palliative therapy)
6	Treatment date	Confirm treatment date
7	Dosage	Confirm cumulative dose if the upper limit of the total dose is set for a drug
8	Infusion rate	Special caution should be exercised for a drug with a different infusion rate between the initial treatment and second and subsequent treatment courses
9	Duration	Confirm whether an anticancer drug exceeds the upper limit of the dosing duration
10	Dose interval	Confirm the reason why a rest period is shorter than the specified period
11	Premedication	Confirm whether all premeditations described in the package inserts are prescribed according to anticancer drugs
12	Latest treatment history	Confirm whether a regimen is performed on a patient to prevent a regimen error (e.g., the use of a previous treatment regimen for the initial treatment)
13	Laboratory test	Confirm examination date (if there is an institution-specific rule, e.g., within the last several months, confirm whether the examination is performed within that period)
14	Urinalysis	
15	Allergy history	Confirm whether a drug used in a regimen can be administered safely by checking a patient’s allergy history
16	Medical history	Confirm whether a drug used in a regimen can be administered safely by checking the side effects of the drug and a patient’s medical history
17	Concomitant drug	Confirm whether a drug used in a regimen can be administered safely by checking a patient’s concomitant drugs (drug interaction, duplicate medication, and contraindications for co-administration)
18	Oral anticancer drug	Confirm days on therapy, dosage, and dose interval in a regimen that concomitantly uses an oral anticancer drug
19	History of hepatitis B virus	Confirm examination date (if there is an institution-specific rule, e.g., within the last several months, confirm whether the examination is performed within that period) Confirm whether HBV-DNA levels are measured within 1–3 months (periods vary depending on each anticancer drug) if a patient tested positive for HBs antibodies or HBc antibodies

**Table 2:** Background of institutions.

Name	Beds	Electronic Medical Records	No. of Pharmacists	No. of Anticancer Drug Preparation
Hokkaido/Tohoku group A	500	Use	17	2544
Hokkaido/Tohoku group B	342	Use	12	6458
Kanto/Shinetsu Group A	560	Use	19	3603
Kanto/Shinetsu Group B	325	Non-use	11	1836
Kanto/Shinetsu Group C	250	Non-use	10	1890
Kanto/Shinetsu Group D	458	Use	13	4910
Kanto/Shinetsu Group E	350	Use	16	806
Tokai/Hokuriku group A	338	Use	16	3175
Tokai/Hokuriku group B	486	Use	26	3263
Kinki group A	304	Use	16	2099
Kinki group B	320	Use	15	3554
Chugoku/Shikoku group	424	Use	11	3707
Kyusyu group A	400	Use	16	4692
Kyusyu group B	300	Use	11	1481

**Table 3:** Results of the questionnaire.

	Control Group N = 62	Recommended Items Group N = 62	*p*-Value
Are current regimen checks performed by pharmacists useful to ensure safe cancer chemotherapy?	7.2 points	8.2 points	<0.05
Do you have confidence in the current regimen checks performed by pharmacists?	5.4 points	6.2 points	<0.05
Did the 19 recommended items affect the time required for completing a regimen check? (Select one that applies)			
(1) More time was required because the number of confirmation items increased		44 pharmacists	
(2) The number of confirmation items increased; however, the time did not change		15 pharmacists	
(3) The number of confirmation items increased; however, the time was shortened		0 pharmacists	
(4) I was unable to use the 19 recommended items because I did not understand them		3 pharmacists	
Is the distribution of the 19 recommended items to hospitals in Japan meaningful? (Select one that applies)			
(1) It is meaningful for the equalization of knowledge and methodology in cancer care		34 pharmacists	
(2) It is meaningful owing to the absence of a definition of regimen checks in my institution; although, I may not utilize them		8 pharmacists	
(3) Further careful examination of confirmation items is required to obtain a maximal benefit from them		20 pharmacists	
(4) It is not meaningful because I am unable to use them		0 pharmacists	

**Table 4:** The mean time required for the completion of a regimen check, according to the regimen, previous cancer chemotherapy history, cancer chemotherapy cycles, and certification of cancer-related associations.

	Control Group	Recommended Items Group
Total	4:14 SD (±1:50) (N = 345)	6:18 SD (±1:7) (N = 375)
Regimen		
SOX	5:15 (n = 36)	6:18 (n = 62)
FOLFIRI + Bev	4:46 (n = 79)	6:57 (n = 93)
Pembrolizumab	2:54 (n = 121)	5:25 (n = 93)
CBDCA + PEM	3:44 (n = 23)	5:49 (n = 21)
EC	5:20 (n = 19)	7:4 (n = 19)
TC	5:23 (n = 67)	6:28 (n = 87)
Previous history of cancer chemotherapy		
Initial	6:18 (n = 44)	7:33 (n = 30)
2nd and onwards	3:56 (n = 301)	6:11 (n = 345)
Cycles of cancer chemotherapy regimens		
1^st^	6:10 (n = 54)	8:20 (n = 41)
2nd and onwards	3:55 (n = 291)	6:3 (n = 334)
Certification of cancer-related associations		
Certified pharmacist	3:17 (n = 58)	4:54 (n = 55)
Noncertified pharmacist	4:26 (n = 287)	6:32 (n = 320)

SOX:S-1 + oxaliplatin; FOLFIRI + Bev:FOLFIRI + bevacizumab; CBDCA + PEM: Carboplatin + pemetrexed; EC: Epirubicin + Cyclophosphamide; TC: carboplatin + paclitaxel. Time is expressed as the mean.

**Table 5:** Inquiries for a doctor’s prescription order by pharmacists.

	Control Group N = 345	Recommended Items Group N = 375
Number of inquiries for a doctor’s prescription order	27 cases (7.8%)	41 cases (10.9%)
Body weight	1 case (0.3%)	1 case (0.3%)
Dosage	5 cases (1.4%)	9 cases (2.4%)
Infusion rate	0 cases (0%)	1 case (0.3%)
Dose interval	0 cases (0%)	1 case (0.3%)
Premedication	4 cases (1.2%)	7 cases (1.9%)
Blood biochemistry	5 cases (1.4%)	0 cases (0%)
Urinalysis	1 case (0.3%)	8 cases (2.1%)
Concomitant drugs	2 cases (0.6%)	1 case (0.3%)
Oral anticancer drug	1 case (0.3%)	1 case (0.3%)
History of hepatitis B	8 cases (2.3%)	12 cases (3.2%)

## Data Availability

The data presented in this study are available upon request from the corresponding author. The data are not publicly available due to confidential issues.

## References

[1] Mitchell G., Porter S., Manias E. (2021). Enabling sustained communication with patients for safe and effective management of oral chemotherapy: A longitudinal ethnography. J. Adv. Nurs..

[2] von Grünigen S., Geissbühler A., Bonnabry P. (2022). The safe handling of chemotherapy drugs in low- and middle-income countries: An overview of practices. J. Oncol. Pharm. Pract..

[3] Mulac A., Taxis K., Hagesaether E., Gerd Granas A. (2021). Severe and fatal medication errors in hospitals: Findings from the Norwegian incident reporting system. Eur. J. Hosp. Pharm..

[4] Weiss B.D., Scott M., Demmel K., Kotagal U.R., Perentesis J.P., Walsh K.E. (2017). Significant and sustained reduction in chemotherapy errors through improvement science. J. Oncol. Pract..

[5] Raymond G., Kerry P. (2012). Chemotherapy medication errors in a pediatric cancer treatment center: Prospective characterization of error types and frequency and development of a quality improvement initiative to lower the error rate. Pediatr. Blood Cancer.

[6] Kohler D.R., Montello M.J., Green L., Huntley C., High J.L., Fallavollita A., Goldspiel B.R. (1998). Standardizing the expression and nomenclature of cancer treatment regimens. American Society of Health System Pharmacists (ASHP), American Medical Association (AMA), American Nurses Association (ANA). Am. J. Health Syst. Pharm..

[7] Jacobson J.O., Polovich M., Gilmore T.R., Schulmeister L., Esper P., LeFebvre K.B., Neuss M.N. (2012). Revisions to the 2009 American Society of Clinical Oncology/oncology nursing Society chemotherapy administration safety standards: Expanding the scope to include inpatient settings. J. Oncol. Pract..

[8] Ranchon F., You B., Salles G., Vantard N., Schwiertz V., Gourc C., Gauthier N., Guédat M.G., Souquet P.J., Freyer G. (2013). Improving cancer patient care with combined medication error reviews and morbidity and mortality conferences. Chemotherapy.

[9] Schnipper J.L., Kirwin J.L., Cotugno M.C., Wahlstrom S.A., Brown B.A., Tarvin E., Kachalia A., Horng M., Roy C.L., McKean S.C. (2006). Role of pharmacist counseling in preventing adverse drug events after hospitalization. Arch. Intern. Med..

[10] Suzuki S., Uchida M., Sugawara H., Suga Y., Nakagawa T., Takase H. (2023). Multicenter prospective observational study on hospital pharmacist interventions to reduce inappropriate medications. Front. Pharmacol..

[11] Uchida M., Suzuki S., Sugawara H., Suga Y., Kokubun H., Uesawa Y., Nakagawa T., Takase H. (2019). A nationwide survey of hospital pharmacist interventions to improve polypharmacy for patients with cancer in palliative care in Japan. J. Pharm. Health Care Sci..

[12] Díaz-Carrasco M.S., Pareja A., Yachachi A., Cortés F., Espuny A. (2007). Prescription errors in chemotherapy. Farm. Hosp..

[13] Freeman P.R., Curran G.M., Drummond K.L., Martin B.C., Teeter B.S., Bradley K., Schoenberg N., Edlund M.J. (2019). Utilization of prescription drug monitoring programs for prescribing and dispensing decisions: Results from a multi-site qualitative study. Res. Soc. Adm. Pharm..

[14] Hayashi M., Yamatani A., Funaki H., Miyamoto K. (2015). Pharmacoeconomic effect of compliance with pharmacist’s intervention based on cancer chemotherapy regimens: A cohort study. J. Pharm. Health Care Sci..

[15] Suzuki S., Chan A., Nomura H., Johnson P.E., Endo K., Saito S. (2017). Chemotherapy regimen checks performed by pharmacists contribute to safe administration of chemotherapy. J. Oncol. Pharm. Pract..

[16] Demachi K., Suzuki S., Kamata H., Suzuki H., Sugama Y., Ikegawa K., Igarashi T., Kawasaki T., Johnson P.E., Yamaguchi M. (2019). Impact of outpatient pharmacy services collaborating with oncologists at an outpatient clinic for outpatient chemotherapy prescription orders. Eur. J. Oncol. Pharm..

[17] Suzuki S., Sakurai H., Kawasumi K., Tahara M., Saito S., Endo K. (2016). The impact of pharmacist certification on the quality of chemotherapy in Japan. Int. J. Clin. Pharm..

[18] Griffin M.C., Gilbert R.E., Broadfield L.H., Easty A.E., Trbovich P.L. (2016). ReCAP: Comparison of Independent Error Checks for Oral Versus Intravenous Chemotherapy. J. Oncol. Pract..

[19] Ohta T., Suzuki S., Shinohara A., Ohashi Y., Ueki D., Konuma D., Ryushima Y., Udagawa R., Kawasaki T., Yamaguchi M. (2021). Needs of chemotherapy regimen checks produce: From the survey on chemotherapy regimen checks performed by pharmacists in hospitals other than designated cancer hospitals in Japan. Eur. J. Oncol. Pharm..

[20] Kanda Y. (2013). Investigation of the freely available easy-to-use software ‘EZR’ for medical statistics. Bone Marrow Transpl..

[21] Tsutsumi Y., Yamamoto Y., Ito S., Ohigashi H., Shiratori S., Naruse H., Teshima T. (2015). Hepatitis B virus reactivation with a rituximab-containing regimen. World J. Hepatol..

[22] Fukuoka M. (2009). Oncology in cancer care. Jpn. Soc. Intern. Med..

[23] (2020). Ministry of Health, Labor and Welfare. Medical Facility Survey. Tokyo. https://www.mhlw.go.jp/stf/seisakunitsuite/bunya/kenkou_iryou/iryou/johoka/index.html.

[24] Nakashima M., Sugiyama T. (2014). A survey of journal articles related to computerized support systems for cancer chemotherapy in Journal of Japanese Society of Hospital Pharmacists and Japanese Journal of Pharmaceutical Health Care and Science. J. Jap. Soc. Health Care Manag..

[25] Nakashima M. (2015). Studies regarding quality enhancement of affairs by pharmacist and clinical evaluation of cancer chemotherapy. Jpn. J. Pharm. Health Care Sci..

[26] Bubalo J., Warden B.A., Wiegel J.J., Nishida T., Handel E., Svoboda L.M., Nguyen L., Edillo P.N. (2014). Does applying technology throughout the medication use process improve patient safety with antineoplastics?. J. Oncol. Pharm. Pract..

[27] Sato H., Ohira H., Murakami T., Kanetaka Y., Murakami T., Izumi H., Hashimoto Y., Komori H. (2016). Comparison between risk listed in the risk management plan and the product labeling of the drug package insert. Jpn. J. Drug Inform..

[28] Suzuki H., Suzuki S., Kamata H., Sugama Y., Demachi K., Ikegawa K., Igarashi T., Yamaguchi M. (2018). Impact of pharmacy collaborating services in an outpatient clinic on improving adverse drug reactions in outpatient cancer chemotherapy. J. Oncol. Pharm. Pract..

[29] Suzuki S., Horinouchi A., Uozumi S., Matsuyama C., Kamata H., Kaneko A., Yamaguchi M., Okudera H., Tahara M., Kawasaki T. (2020). Impact of outpatient pharmacy interventions on management of thyroid patients receiving lenvatinib. SAGE Open Med..

[30] Suzuki S., Abbott R., Sakurai H., Kawasumi K., Johnson P.E., Tahara M., Yamaguchi M., Saito S., Yee G.C., Endo K. (2017). Evaluation of community pharmacist ability to ensure the safe use of oral anticancer agents: A nationwide survey in Japan. Jpn. J. Clin. Oncol..

